# Robust Metallic Nanolaminates Having Phonon-Glass Thermal Conductivity

**DOI:** 10.3390/ma13214954

**Published:** 2020-11-04

**Authors:** Francisco Alfredo García-Pastor, Josué Benjamín Montelongo-Vega, Marco Vinicio Tovar-Padilla, María Antonia Cardona-Castro, Jaime Alvarez-Quintana

**Affiliations:** 1 Centro de Investigación y de Estudios Avanzados del Instituto Politécnico Nacional Unidad Saltillo, Avenida Industria Metalúrgica No. 1062. Parque Industrial, Ramos Arizpe 25900, Coahuila, Mexico; jmontelo@ternium.com.mx (J.B.M.-V.); antonia.cardona@cinvestav.edu.mx (M.A.C.-C.); 2 Ternium Mexico S.A. de C.V, Planta Pesquería, Carretera a Pesquería Km. 15, Ejido La Victoria, Los Ramones 66650, Mexico; 3 Centro de Investigación en Materiales Avanzados S. C. Unidad Monterrey, Alianza Norte # 202, Autopista Mty-Aeropuerto Km.10, Apodaca 66600, Nuevo León, Mexico; opira666@gmail.com; 4 Genes-Group of Embedded Nanomaterials for Energy Scavenging, CIMAV-Unidad Monterrey, Apodaca 66600, Nuevo León, Mexico

**Keywords:** nanoscale heat transfer, metallic multilayer composites, high performance thermal barriers, thermal boundary resistance, accumulative roll bonding

## Abstract

Heat transfer phenomena in multilayer structures have gained interest due to their promising use in thermal insulation and thermoelectricity applications. In such systems, nanostructuring has been used to introduce moderate interfacial density, and it has been demonstrated that interfacial thermal resistance plays a crucial role in reducing thermal conductivity κ. Nevertheless, the main constraint for actual applications is related to their tiny size because they are extremely thin to establish appreciable temperature gradients. In this work, by severe plastic deformation process of accumulative roll bonding (ARB), a 250 µm thick Cu-Nb multilayer containing more than 8000 interfaces with periods below 40 nm was obtained, enabling the production of bulk metallic nanolaminates with ultralow κ. Multilayers present an ultralow κ of ∼0.81 W/mK at 300 K, which is 100 times smaller than its Cu-Nb bulk counterpart, and even lower than the amorphous lattice limit for the Cu-Nb thin film system. By using electron diffusive mismatch model (EDMM), we argue that both electrons diffusively scattering at interface and those ballistically crossing the constituents are responsible for heat conduction in the Cu-Nb multilayers at nanoscale. Hence, ARB Cu-Nb multilayers are intriguing candidate materials which can prove avenues to achieve stable ultralow κ thermal barriers for robust applications.

## 1. Introduction

Decreased efficiencies in energy conversion systems, as well as failure commonly found in power microelectronic circuits are the main motivation towards the development of new thermal dissipation and insulation materials embedded in the operating components. In this sense, multilayer structures have gained interest due to their promising use as tunable thermal conductivity materials via their interfacial thermal resistance [1,2]. Nevertheless, multilayered architecture-based devices utilizing 2D layered materials have opened a world of chances and failures. For instance, interfaces between solid layers in nanoelectronics are undesirable because they contribute significantly to the overall thermal resistance, which is a bottleneck for the further advancement of such devices. Thermal management in these devices has become problematic because faster and denser circuits are being packaged; as a consequence, localized areas of high heat flux are dominating the performance of electronics at both chip and board level in current nm-technologies [3,4,5,6]. On the other hand, the recent boost of research in thermoelectricity and thermal protection is closely linked to recent advances in phonon engineering via materials nanostructuring. In such applications, very low thermal conductivity materials are desirable to better engineer those systems [7,8,9,10,11]. To the present day, extensive experimental and theoretical investigations of thermal transport across metal–dielectric interfaces have been conducted to gain further understanding of the physics behind nanoscale thermal transport. For instance, Costescu et al. reported an ultralow κ of ∼0.6 W/mK in W/Al_2_O_3_ nanolaminates with a high interface density [12]. Kim et al. observed a lower value of κ ∼0.3 W/mK in Ge_2_Sb_2_Te_5_/ZnS:SiO_2_ multilayers [13], and Chen et al. in Au/Si multilayers found a value as low as 0.33 W/mK via solids with highly dissimilar Debye temperatures [14]. In those systems, due to the metal region being thick compared with the energy carrier mean free paths, thermal energy transfer is mainly rendered by the electron–phonon coupling at the interface. Conversely, Goodson et al. reported a κ of ∼1.2 W/mK in Mo/Si multilayers with periods below 10 nm; in this case, the small thickness of the metal film inhibits the electron–phonon coupling, which suppresses the electron contribution to conduction in the film-normal direction and renders phonons the dominant heat carriers [15]. Evidently, while the mechanisms related to cross-plane heat conduction in multilayers have been studied in detail previously, the main constraint for actual applications is related to their tiny size. They are too thin to establish appreciable temperature gradients and to withstand large heat fluxes. For instance, in mechanical compression refrigeration systems, thermal insulation between the interior chamber and the steel laminate exterior cabinet occurs via a thick polyurethane panel [16]. It is possible that in high-specification systems and niche applications, a thermal insulation material with adequate mechanical properties and thermal stability as part of the exterior cabinet would be more convenient. Following this idea, processing of a sample with hundreds or thousands of interfaces on a wide area with significant thickness via habitual layer-by-layer deposition techniques such as radio frequency (RF) sputtering, E-beam evaporation, atomic layer deposition (ALD), molecular-beam epitaxy (MBE), or any other would be impractical. In this work, we present a rational approach to achieve ultralow thermal conductivity bulk metallic multilayers via ultrahigh content of interfaces with potential in robust structural applications as part of the operating components. In contrast to sputtering or atomic layer deposition techniques, which only yield extremely thin specimens, we follow a processing route to obtain bulk nanolaminates by severe plastic deformation process of accumulative roll bonding (ARB) in bimetallic strips. Following this route, a 250 μm thick Cu-Nb multilayer containing more than 8000 interfaces with periods below 40 nm has been obtained with a value of κ ∼0.81 W/mK at room temperature. Such value is lower than what is predicted by the amorphous limit model developed by Cahill et al. for the Cu-Nb thin film system [17]. In fact, it is well below the value of κ for amorphous dielectrics such as SiO_2_ [17]. Moreover, by using the EDMM, it is argued that both electrons diffusively scattering at the interface and those ballistically crossing the constituents are responsible for heat conduction in the Cu-Nb multilayers at nanoscale.

## 2. Materials and Methods

**Figure 1:**
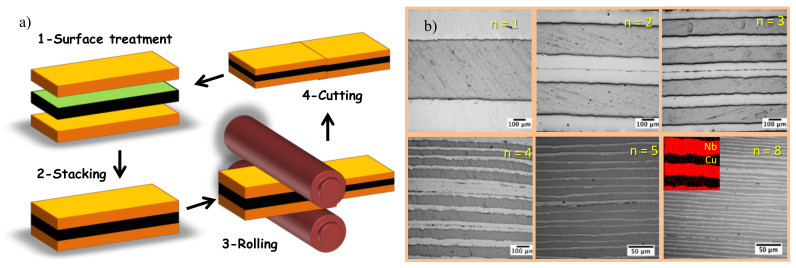
(**a**) Schematic of the ARB process, and (**b**) Optical images for samples with different number of ARB iterations. Inset shows the energy-dispersive-spectroscopy (EDS) chemical analysis of the Nb-Cu multilayers.

Copper and niobium are immiscible in the solid range, as seen in the phase diagram for this system [18]. This limitation arises from the differences in crystal structure between the two metals, as well as the large mismatch between the constituents’ atomic radii. Therefore, such materials are good candidates for the development of bulk nanolamellar Cu-Nb composites for robust thermal barrier applications. Starting materials consist of single-phase laminates of commercial purity Cu and Nb which are degreased, wire-brushed, and stacked. Iterating these steps increases yields to an exponential increase of the number of layers while decreasing layer thickness. A schematic representation of the general ARB process is shown in Figure 1a. ARB processing was carried out in a rolling mill with a maximum separating force of 20 metric tons, equipped with D2 steel rollers of 10 cm in diameter, following the procedure as described in reference [19]. Before stacking, surfaces of Cu-Nb-Cu sheets of 500, 1000 and 500 microns thick, respectively were brushed and cleaned using acetone in an ultrasonic bath. Then, stacks were preheated in an inert atmosphere furnace at 700 °C for 4 min, and the rolling linear speed was set at 5 × 10^−3^ m/s. After each ARB pass, the bonded laminate was cut transversally in half using a mechanical shear. The two halves were then subjected to repetitive process of cleaning, stacking and rolling until obtaining the multilayers with ultrahigh content of interfaces.

In Figure 1b, optical images of the multilayers after eight sequential ABR processes are shown. Evidently, after each ABR stage, the number of interfaces *N* is increased following the power law *N* = 2*^n^* where *n* stands for the process number. Based on this, the optical image with *n* = 8 presents a transversal section of the sample containing 256 interfaces, which is a considerable number of interfaces. Inset shows the EDS (Oxford Instruments plc, Abingdon, Oxfordshire, UK) chemical analysis of the multilayers, Cu and Nb were identified as alternating layers. Although ARB has been applied to several different bimetal systems [20,21,22,23], the Cu-Nb system offers negligible solid solubility between the two phases. These characteristics result in excellent microstructural stability during ARB processing, allowing production of bulk metallic nanolaminates. In this sense, intense investigations have been made into determining the effects of interface character on behavior of ARB Cu-Nb composites in terms of mechanical behavior and stability when exposed to elevated temperature [24]. However, one logical extension of the works performed here is study of Cu-Nb nanolaminates intended for heat protection, where simultaneously high strength, thermal stability, and very low thermal conductivity will be key engineering requirements for next generation of robust thermal barrier materials. In this work, tensile strength was measured using small-scale notched tensile samples, with a cross-sectional area of 2 mm^2^. A Deben Microtester (Deben UK, Ltd., Woolpit, Bury St. Edmunds, UK) equipped with a 2 KN load cell was used for tensile testing. Thermal response of the samples has been carried out via the hot-plate method. To prevent uncertainties, the measurement system was put inside of a conventional vacuum system to avoid heat loss by convection. Additionally, a radiation shield is used in order to protect the equipment from thermal fluctuations coming from the environment. Details of thermal measurements are given in the Supplementary Materials.

## 3. Results

**Figure 2:**
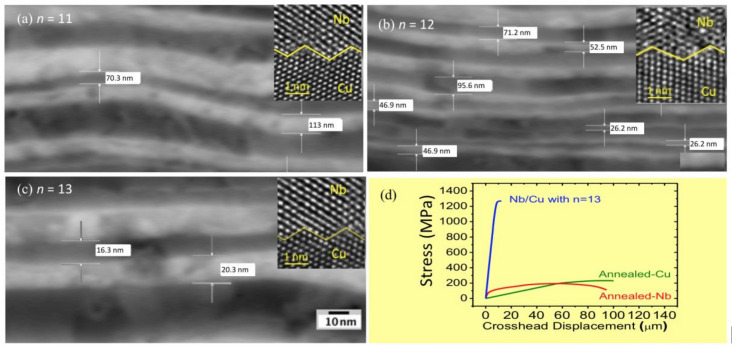
(**a**) HRTEM image of the Nb-Cu multilayers with *n* = 11, (**b**) *n* = 12 and (**c**) *n* = 13 ARB iterations. (**d**) UTS for sample with *n* = 13 ARB iterations as compared with their annealed constituents. Insets shows HRTEM images of the multilayers at the interface.

Figure 2 shows scanning electron microscopy (SEM) images for samples with *n* = 11, *n* = 12 and *n* = 13, iterations, which theoretically correspond to *N* = 2048, *N* = 4096 and *N* = 8192 interfaces, respectively. It can be seen that there are well-defined interfaces formed by continuous layers of some nanometers in thickness, e.g., in sample with *n* = 13, a period below 40 nm is identified. Insets present high-resolution transmission electron microscopy (HRTEM) images at the interfaces. Images indicate that both Cu and Nb retain their bulk stable crystal structures, resulting in a stacking sequence of Cu in face-centered cubic (FCC) structure and Nb in body-centered cubic (BCC) structure forming highly incoherent Cu-Nb interfaces with few interfacial dislocations. Previous experimental investigations confirm that there is no mixing at the Cu-Nb interface up to 873 K; the interface texture is stable as well up to 973 K, where a Kurdjumov–Sachs orientation relationship ({112}Cu || {112}Nb) is the most thermally stable [24,25], which is an essential requisite to obtain highly stable interfacial thermal resistances. Besides, interface modeling based on molecular dynamics has confirmed such incoherence and low interfacial dislocation in faceted FCC(Cu)/BCC(Nb) interfaces [26]. Obviously, synergy between thermal and mechanical properties is indispensable for robust stable thermal barriers as part of the operating components. Metallic-based multilayered nanocomposites are recognized for their increased plastic flow strength, increased ductility, and enhanced fatigue-failure resistance in comparison to their coarse-grained constituents [27]. Compared to either pure Cu or Nb which display an ultimate tensile strength (UTS) of just 210 and 150 MPa, respectively in the annealed condition, the 13-iterations ARB-processed composite exhibits a 5-fold increase in UTS, reaching just under 1300 MPa of tensile strength, as shown in Figure 2d. Thus, compared to the bulk constituent materials, the nanocomposite is mechanically stronger, which is particularly relevant for potential in robust structural applications. The dramatic increase in UTS is mainly related to the stability of Cu-Nb interfaces which act as effective barriers to slip transmission [28].

**Figure 3:**
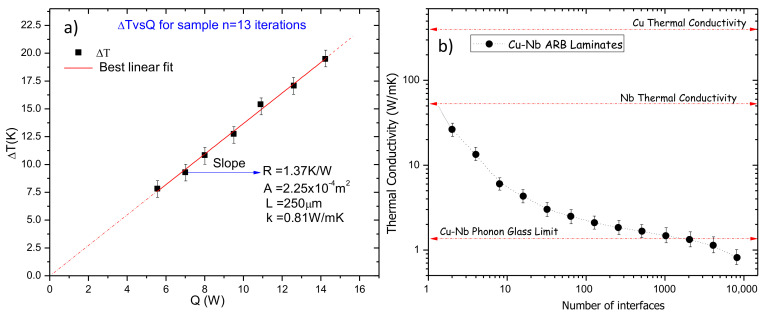
(**a**) Δ*T*–*Q* transfer curve for sample with *n* = 13, and (**b**) κ for samples *n* = 1 to *n* = 13.

Previous studies suggest that interfaces between metallic materials with considerable differences in their lattice constants and structures would possess a high interfacial thermal resistance [27,28,29,30,31], and if combined with high interface content via multilayers, one would expect a very low κ. Figure 3a shows the Δ*T*–*Q* transfer curve for sample with *n* = 13 iterations. Using the geometrical parameters, and the slope which represents the thermal resistance, a value of κ ∼ 0.81 W/mK was obtained. By a similar procedure, the κ of the multilayer samples from *n* = 1 to *n* = 12 was measured.

Figure 3b shows the thermal conductivity of the ARB Cu-Nb composites as a function of the number of interfaces. Evidently, as the number of interfaces increases, the thermal conductivity decreases drastically; this behavior makes sense because the overall interfacial resistance increases as the number of interfaces increases. According to the literature, values of *k_Cu_* ≈ 400 W/mK and *k_Nb_* ≈ 54 W/mK are reported for bulk Cu and Nb, respectively. Therefore, by using the thermal resistor model, the effective thermal conductivity neglecting interfacial resistance effects for a two-segment slab is given by

(1) $$k_{slab}=\frac{2k_{1}k_{2}}{\left(k_{1}+k_{2}\right)}$$

where *i* defines the metal. Based on Equation (1), an effective thermal conductivity value of around 95 W/mK for the bulk Cu-Nb structure is expected. Nevertheless, for *n* = 1 the experimentally obtained value of κ drops drastically to 26.5 W/mK, thus significant thermal resistance effects at the interface are present. Under a physical context, contact between two surfaces occurs in a few discrete points because of the asperities and roughness present in actual surfaces. As a result, heat flux will tend to pass through the small contact points and will avoid the interstices. Depending on the surfaces’ roughness in contact, temperature discontinuity at the interface between two dissimilar solids can be promoted either by the thermal contact resistance (TCR), usually with very rough surfaces whose root-mean-square roughness is greater than 50 μm [31], or by the interfacial thermal resistance (ITR) with surfaces roughness ranging from some nanometers to several tens of nanometers [2,3]. Following this, the effective thermal conductivity of a two-segment slab taking into consideration both effects is given by

(2) $$k_{ef}^{-1}=k_{slab}^{-1}+\frac{R_{ITR}}{t_{1}}+\frac{R_{TCR}}{t_{2}}$$

where *R_ITR_* and *R_TCR_* stand for the interfacial and contact thermal resistances, and *t_i_* defines the period thickness.

Extensive experimental and theoretical studies have been carried out in the past to explain the ITR through interfaces, but the acoustic mismatch model (AMM) and the diffusive mismatch model (DMM) are the two basic models [32,33]. The AMM assumes that incident phonons at an interface undergo specular reflection or transmission and are governed by continuum mechanics, whereas in the DMM, the probability of phonon transmission to either side of the interface depends on the ratio of the density of phonon states. Pursuant to DMM, the ITR spans in a range from 10^−7^ to 10^−9^ m^2^·K/W [32,33,34]. Hence, only multilayers with period length below 10^−9^ m are influenced significantly in their overall thermal conductivity by the ITR in accordance with Equation (2).

**Figure 4:**
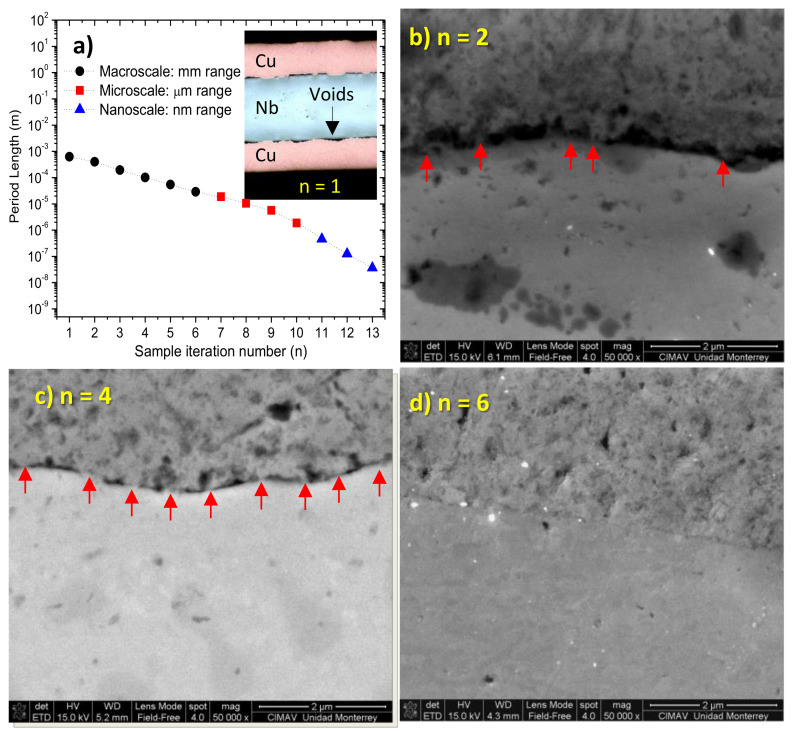
(**a**) Period length vs ARB iteration number. Inset shows sample with *n* = 1 iteration, contact points and voids are evident at the interfaces. Samples with (**b**) *n* = 2, (**c**) *n* = 4, (**d**) *n* = 6 ARB iterations, respectively.

Figure 4a shows the period length as a function of the iteration number *n* for the ARB Cu-Nb composites. It can be seen that period length approximately spans over three scales: mm range for 1 ≲ *n* ≲ 6, μm range for 7 ≲ *n* ≲ 10 and nm range for 11 ≲ *n* ≲ 13. Thus, only samples for *n* ≳ 11 must experience significant ITR effects, which means that the second term in the right side of Equation (2) tends to zero in the mm and μm range. Therefore, the drastic reduction observed in κ for the ARB Cu-Nb composites with *n* ≲ 10 is linked to the TCR term in Equation (2). The inset in Figure 4a shows an optical image for the sample with *n* = 1 iteration, contact points and voids are evident at the interfaces. Moreover, Figure 4b–d shows SEM images for samples with *n* = 2, *n* = 4 and *n* = 6 iterations, respectively. Clearly, when pressure on the interface is increased via ARB process, the points in contact are deformed and they increase both in size and number, as highlighted by the red arrows in the images for samples with *n* = 2 and *n* = 4. Hence, the interfaces between metals become gradually in total contact because of the reduction of the surface’s roughness, as shown for a sample with *n* = 6.

## 4. Discussion

**Figure 5:**
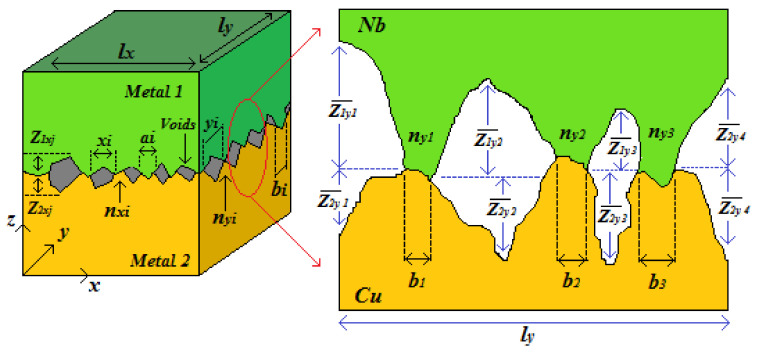
Illustrative model of an interface between two contacting metallic surfaces for samples with period lengths in the mm range, i.e., 1 ≲ *n* ≲ 6. In the figure, *n_xi_*, *a_i_*, *x_i_* and *n_yi_*, *b_i_*, *y_i_*, represent the number of contact points, size of the contact points and size of the voids in the *x* and *y* directions, respectively. $\bar{Z_{ixj}}$ and $\bar{Z_{iyj}}$ are the void average height in the *x* and *y* directions, respectively.

To gain a quantitative understanding of these results, we suggest the following model. Figure 5 shows an illustrative representation of actual metallic interfaces. It is considered that, for most contacts, void height is relatively smaller than its width. Because of this relative size, it is possible to neglect radial heat convection as well as heat conduction across the voids. Thus, the thermal contact resistance *R_TCR_* associated with this type of interface will depend on the thermal conductivity of the metals as well as the thermal conductivity of the fluid filling the voids, and it can be estimated using the method proposed by Rohsenow [35] as:

(3) $$\frac{1}{R_{TCR}}=\frac{\left(\frac{k_{f}}{\delta_{1}+\delta_{2}}\right)\left[\left(1-\alpha^{2}\right)C+1.1\alpha f\left(\alpha \right)\left(\frac{1}{k_{1}}+\frac{1}{k_{2}}\right)\right]+4.26\alpha \sqrt{n_{p}}}{\left(1-\alpha^{2}\right)\left[1-\left(\frac{k_{f}}{\delta_{1}+\delta_{2}}\right)\left(\frac{\delta_{1}}{k_{1}}+\frac{\delta_{2}}{k_{2}}\right)\right]C}$$

The equation for *R_TCR_* presented in Equation (1) is a sum of two terms. Within the square brackets in the numerator (effectively, the first term) is an expression representing heat flow through the voids. Heat flow through the metallic contacts is considered in the second term. *k_i_* represents the thermal conductivity of the metals forming the interface, and *k_f_* the thermal conductivity of the fluid in the void. Likewise, *α* is the squared root of the ratio of the real area of contact *Ac* to the total contact area *A*. Besides, in Equation (3), *δ_i_* is a parameter that expresses the equivalent idealized gap thickness in terms of the average heights $\bar{Z_{ixj}}$ and $\bar{Z_{iyj}}$ of the voids of actual surfaces in the *x* and *y* direction respectively, as shown in Figure 5. The subscript *i* refers to metal 1 or 2, and *j* refers to the number of the void. The number of contact points per unit area *n_p_* is obtained by dividing the product of the number of contacts on each pair of profiles in the *x* and *y* directions, i.e., *n_xi_n_yi_* over an area defined by *l_x_l_y_*. The term *C* is a factor which is only used to compact Equation (3), and it is given by

(4) $$C=\frac{1+4.26\sqrt{n_{p}}\left(\frac{\delta_{1}}{\alpha }\right)}{k_{1}}+\frac{1+4.26\sqrt{n_{p}}\left(\frac{\delta_{2}}{\alpha }\right)}{k_{2}}$$

**Figure 6:**
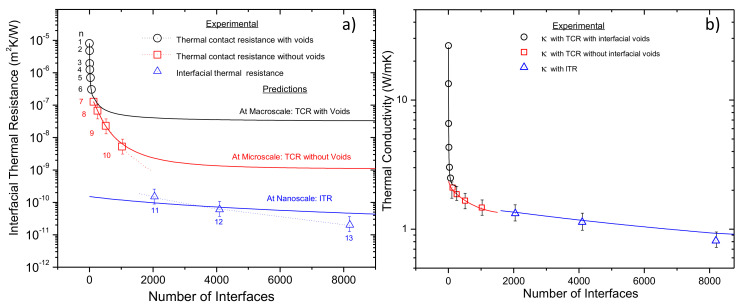
(**a**) Interface-dependent thermal resistance at interface, and (**b**) Interface-dependent thermal conductivity for ARB samples. In both plots, solid lines are the calculated *R_eITR_*, *R_TCR_* and κ values which are reasonably consistent with the experimental data.

Finally, with these quantities α, *n_p_* and *δ_i,_* the magnitude of *R_TCR_* can be predicted from Equation (3). Experimental thermal resistance at interface for the whole set of samples is shown in Figure 6a as open symbols. Such values were extracted from the effective thermal conductivity data shown in Figure 3 along with Equation (2). The black solid line and open circle symbols represent the modeling and experimental data respectively for samples with 1 ≲ *n* ≲ 6 iterations. Evidently, beyond *n* ≳ 7, the model given by Equation (3) deviates significantly. It is worth mentioning that because voids at the interface diminish as the iteration number increases, it was possible to accomplish reasonably good modeling results only for samples with *n* ≲ 6.

The problem with Equation (3) is related to the function *f*(*α*); for practical purposes, the ratio of the squared root of the effective area of contact to the total contact area must be *α* ≲ 0.1. Under this condition, *f*(*α*) ≈ 1, otherwise, *f*(*α*) drops drastically well below 1. The physical meaning of *f*(*α*) ≪ 1 implies that the effective area of contact to the total contact are very similar, thus *α* ≈ 1, i.e., no more voids or a very high contact points density; hence, the model fails in such a situation. To overcome such condition, the first term in Equation (3) can be neglected since flow across the voids is neglected as well. By using such math artifice, Equation (3) becomes independent of *f*(*α*), and the model can be applied for samples with periods at the microscale where the interfaces have much better contact, i.e., 7 ≲ *n* ≲ 10. In Figure 6a, square open symbols and a red solid line represent the modeling and experimental data, respectively, for samples with 7 ≲ *n* ≲ 10 iterations. Evidently, beyond *n* ≳ 11, the model again deviates significantly, and it is unable to predict accurately the contact resistance for samples with periods at the nanoscale, i.e., 11 ≲ *n* ≲ 13. The detailed procedures of calculations of the *R_TCR_* at the macro/microscale regime are given in the Supplementary Materials (Pages 5–11, Equations (1)–(10)).

At the nanoscale regime, when the films’ thicknesses are relatively larger than the electron mean free path, pure diffusion is adequate to model thermal transport inside them. Therefore, assuming perfect contact between the two metals, it is also possible to assume that electrons are in equilibrium on each side of the interface, and just at the interface, electrons will be scattered following a probability that depends solely on the properties of the metals. Nevertheless, when the films’ thicknesses are comparable with or smaller than the electron mean free path, thermal transport must be modeled as purely ballistic. If we take into consideration that the average period length for Cu-Nb composites with *n* ≳ 11 ranges from 400 nm to 40 nm, while Cu and Nb are both free-electron metals with mean free paths of ∼86 and ∼8 nm, respectively, then the electron diffusive mismatch model EDMM assumptions are adequately describing electron transport across the aforementioned interfaces. In the EDMM approach [30], the interfacial thermal resistance is given by

(5) $$\frac{1}{R_{ITR}}=\frac{1}{4}\left(\frac{Z_{1}Z_{2}}{Z_{1}+Z_{2}}\right)$$

where $Z_{i}=C_{ei}v_{Fi}$; here, $C_{ei}$ is the electronic heat capacity and $v_{Fi}$ is the electron Fermi velocity of the metal on side *i*. Evidently, electrons move ballistically from the copper side to the niobium side, where then they scatter diffusively depending on the surface roughness. Hence, the effective thermal interface resistance is thus the sum of the contribution of both the electrons ballistically traveling across the Cu-Nb system and those diffusively scattering at the interface. Therefore, the effective ITR is given by

(6) $$\frac{1}{R_{eITR}}=\frac{1}{R_{ITR-B}}\left(\beta \right)+\frac{1}{R_{ITR-D}}\left(1-\beta \right)$$

where $\beta =e^{\left(-d/\lambda_{e}\right)}$ is a term derived from ballistic diffusive equations and describes the exponential decay of heat flux across interface [36]. *R_(ITR−B)_* and *R_(ITR−D)_* stand for the ballistic and diffusive ITR contributions, respectively. Values of the parameters and calculations of the *R_eITR_* are given in the Supplementary Materials (Pages 11–12, Equations (11)–(19)). In Figure 6a, blue open triangle symbols show the experimental data of Cu-Nb multilayers with nanometric period lengths. Such data are in reasonable agreement according to the EDMM results given by the blue solid line. Clearly, from Figure 6a, the thermal resistance at the interface depends on the scale regime. At macro/microscale regimes *R_TCR_* lies between 10^−6^ and 10^−8^ m^2^·K/W; whereas at nanoscale regime, *R_TCR_* lies between 10^−9^ and 10^−11^ m^2^·K/W. By examining the literature, at macro/microscale, *R_TCR_* in metal–metal interfaces depends strongly on several factors such as surface roughness, asperity and pressure. In general, as the contact pressure increases, *R_TCR_* decreases, and as the roughness of the surface decreases, *R_TCR_* decreases. Fletcher et al. reported *R_TCR_* values between layers of stacked aluminum alloys ranging from 10^−4^ to 10^−6^ m^2^ K/W under moderate contact pressure, but lower values can be accomplished for smooth surfaces under high pressures [37]. At the nanoscale, multilayer metallic composites in which electrons in constituent metals have considerable different mean free paths may provide a quantitative comparison with our *R_eITR_* experimental values. Wilson and Cahill reported a *R_ITR_* ≈ 8.2 × 10^−11^ m^2^·K/W across Pd-Ir interfaces at room temperature [29], whereas Gundrum et al. reported a *R_ITR_* ≈ 2.7 × 10^−10^m^2^·K/W across Cu-Al interfaces [30]. In the present work, for Cu-Nb composites with *n* = 11, *n* = 12 and *n* = 12, the experimental interfacial thermal resistances are 1.5 × 10^−10^ m^2^·K/W, 6.2 × 10^−11^ m^2^·K/W and 2.2 × 10^−11^ m^2^·K/W, respectively. Thus, our experimental results are in reasonable agreement with previously reported metallic interfaces, but with significantly larger (250 micrometers versus 1–2 micrometers in [30]) and more mechanically robust samples. Besides, for the sample with *n* = 13 which corresponds to a period length below 40 nm, the EDMM approach predicts a value of 5.4 × 10^−11^ m^2^·K/W, hence experimental and predicted values are similar. By using the predicted *R_TCR_* and *R_eITR_* data along with Equation (2), the effective thermal conductivity of the Cu-Nb composites has been estimated. Figure 6b shows the modeling results, and experimental data as solid lines and open symbols, respectively. It is worth highlighting the ultra-low thermal conductivity value of *k_(Cu-Nb)_* ≈ 0.81 W/mK obtained for the sample with *n* = 13 iterations. At this level, the term “ultra-low” is used to describe a κ value lower than that predicted by the minimum κ model presented by Cahill et al. [17] Therefore, the existence of a lower limit to the thermal conductivity of disordered crystals based on the idea that lattice vibrations in those solids are essentially the same as those of an amorphous solid can be approximated by

(7) $$k_{min}=0.403k_{B}n_{a}^{2/3}\left(2v_{T}+v_{L}\right)$$

where *ν_T_* and *ν_L_* are the transversal and longitudinal speeds of the sound, respectively, *n_a_* is the number of density of atoms and *k_B_* the constant of Boltzmann. For comparison, Table 1 shows the minimum thermal conductivity values predicted by Equation (7), as well as the reported experimental thermal conductivity for amorphous bulk materials [17]. Clearly, the thermal conductivity of 0.81 W/mK at room temperature for the sample with *n* = 13 is even lower than the 0.95 W/mK predicted for the amorphous lattice limit of the Cu-Nb thin film system. In fact, it is determined to be well below that of the amorphous dielectrics like Al_2_O_3_ or SiO_2_, and about 100 times lower than the Cu-Nb system with *n* = 0 iterations.

## 5. Conclusions

In conclusion, we obtained by severe plastic deformation process of accumulative roll bonding (ARB) a 250 μm thick Cu-Nb multilayer containing more than 8000 interfaces with periods below 40 nm. Such a result enables the production of bulk metallic nanolaminates with ultralow thermal conductivity values of around 0.81 W/mK at room temperature which is much lower than that of the amorphous dielectrics like SiO_2_. The large differences in electron mean free paths in Cu and Nb along with the thermodynamic immiscibility of these materials make them an ideal system to study the role of electron thermal transport properties on thermal conductance across metallic interfaces. We report an experimental value of 2.2 × 10^−11^ m^2^·K/W for ITR between Cu and Nb interfaces at room temperature for the sample with 40 nm period length. Although, *R_ITR_* values obtained by the EDMM model do not match accurately the estimated *R_ITR_* values they give an approach about the trend of the *R_ITR_* as n increases. It is well known that such model is limited to single crystal metallic thin films, and this could be the main reason for the lack in the data fit.

The results presented are adequately described using the electronic diffuse mismatch model EDMM. This indicates that the EDMM mechanisms are acceptable for describing electron transport through the Cu-Nb interfaces. Besides, our results are comparable to previously reported values on Al-Cu and Pd-Ir interfaces. In contrast to similar low-thermal conductivity laminates such as the ones reported in References [12,14,30], the ARB processing route yields bulk nanolaminates with remarkable mechanical strength. Not only that, but the thickness is two orders of magnitude larger in the processed samples reported in this paper, compared to samples obtained by sputtering or atomic layer deposition. Therefore, the combination of microstructural and thermal stability, high strength, and ultralow thermal conductivity make ARB metallic nanolaminates intriguing candidate materials for structural applications where the current thermal barriers based on multilayers only available in thin-film form would prove inadequate.

## Figures and Tables

**Table 1:** Predicted values for the lower limit to thermal conductivity of amorphous materials.

Sample	*n* (10^28^ m^−3^)	*v*_T_ (m/s)	*v*_L_ (m/s)	*K*_min_ (W/mK)	*K*_exp_ (W/mK)
SiO_2_	6.63	3740	5980	1.21	1.35
Al_2_O_3_	10.89	5800	9900	2.71	2.76
Cu	8.47	3720	4720	1.3	-
Nb	5.56	2092	5068	0.76	-
a-Cu/Nb System	-	-	-	0.95	
Cu-Nb (n = 0)	-	-	-	-	92.5
Cu-Nb (n = 13)	-	-	-	-	0.81

## Supplementary Materials

The following are available online at https://www.mdpi.com/1996-1944/13/21/4954/s1, Figure S1: (**a**) Schematic of the ARB process, and (**b**) Optical images for samples with a different number of ARB iterations. Inset shows the EDS chemical analysis of the Nb-Cu multilayers. Figure S2: (**a**) TEM image of the Nb Cu multilayers with n = 11, (**b**) n = 12 and (**c**) (n = 13 ARB iterations. (**d**) UTS for sample with n = 13 ARB iterations as compared with their annealed constituents. Insets shows HRTEM images of the multilayers at the interface. Figure S3: (**a**) Illustrative and (**b**) actual experimental set up for thermal conductivity measurements. Figure S4: ΔT–Q transfer curve for reference sample. Figure S5: ΔT–Q transfer curve for sample with n=13 iterations. Figure S6: Illustrative model of an interface between two contacting metallic surfaces for samples with period lengths in the mm range, i.e., 1≲n≲6. In the figure, $n_{xi}$, $a_{i}$, $x_{i}$ and $n_{yi}$, $b_{i}$, $y_{i}$, represent the number of contact points, size of the contact points and size of the voids in the *x* and *y* direction, respectively. Figure S7: (**a**) Period length vs. iteration number *n* for the ARB Cu-Nb composites, and (**b**) *n* = 2, (**c**) *n* = 4, (**d**) *n* = 6 present the interface evolution. Figure S8: Size of the contact points in the *x* and *y* directions, respectively. Figure S9: Average heights $\bar{Z_{ixj}}$, and $\bar{Z_{iyj}}$ of the voids of actual surfaces in the *x* and *y* directions, respectively. Figure S10: Size of the voids in the *x* and *y* directions, respectively. Figure S11: Number of contact points per unit area *n*. Figure S12. Size-dependent thermal conductivity for Cu and Nb polycrystalline films. Solid lines represent the results obtained from model reported in reference [12], whereas solid points represent the existing experimental data for Cu. Table S1: Parameters used for sample with n = 2 iterations. Table S2: Calculated parameters for Cu and Nb using the free electron model. Table S3: Calculated data for the interface thermal resistance between copper and niobium interfaces. Table S4: Effective thermal interface resistance calculations for samples with n = 11, n = 12 and n = 13 iterations. Table S5. Predicted values for the lower limit to thermal conductivity of amorphous materials. Table S6. Summary of typical uncertainty of measuring parameters. Determination of the interface physical properties for actual surfaces such as real area in contact, the number of contact points per unit area, and the average thickness of the void gaps via image analysis, as well as calculation of the thermal contact resistance *R_TCR_*, pages 5–11, Equations (1)–(10). Determinations of the materials properties (Cu and Nb) such as electron mean free path, density of states, Fermi velocity, and transmission coefficient, as well as calculation of the interfacial thermal resistance *R_ITR_*, pages 11–12, Equations (11)–(19). Detailed discussion of the EDMM and TCR approaches, as well as calculations of the minimum thermal conductivity, pages 13–14. Measurement uncertainty assessment, pages 15–16. References [38,39,40,41,42] are cited in the supplementary materials.

## Nomenclature

ARB	accumulative roll bonding
SPD	severe plastic deformation
EDMM	electron diffusive mismatch model
*k*	thermal conductivity
N	number of interfaces
*n*	number of ARB passes
HRTEM	high-resolution transmission electron microscopy
Q	heat
*k_Cu_*	copper thermal conductivity
*k_Nb_*	niobium thermal conductivity
*k_slab_*	two-segment bimetallic thermal conductivity
*R_ITR_*	interfacial thermal resistance
*R_TCR_*	thermal contact resistance
AMM	acoustic mismatch model
DMM	diffusive mismatch model
*k_i_*	thermal conductivity of the metals forming the interface
*i*	subscript to refer to either metal 1 or 2
*k_f_*	thermal conductivity of the fluid in the void
*α*	squared root of the ratio of the real area of contact to the contact area
*Ac*	real area of contact
A	total contact area
*δ_i_*	equivalent idealized gap thickness in terms of average height
$\bar{Z_{ixj}}$, $\bar{Z_{iyj}}$	average height of voids
*j*	subscript index for void number
*n_p_*	number of contact points per unit area
*C_ei_*	electronic heat capacity of metal *i*
v_Fi_	electron Fermi velocity of metal *i*
β	term describing the exponential decay of heat flux across the interface
*R_ITR-B_*	ballistic ITR contribution
*R_ITR-D_*	diffusive ITR contribution
*k_min_*	thermal conductivity lower limit
*ν_T_*	transversal speed of sound
*νL*	longitudinal speed of sound
*n_a_*	number of density of atoms
*k_B_*	constant of Boltzmann
*R_eITR_*	effective interfacial thermal resistance
*λ_e_*	effective mean free path of electrons
d	half metallic lamellae thickness
