# Supplementary material for: Robust Metallic Nanolaminates Having Phonon-Glass Thermal Conductivity
Source: Materials (Basel). 2020 Nov 4;13(21):4954. doi: 10.3390/ma13214954 (PMC7662775; doi:10.3390/ma13214954)
Supplement: Supplementary file 1 [file materials-13-04954-s001.pdf]

materials

MDPI

Supporting Information

# Robust Metallic Nanolaminates Having Phonon-Glass Thermal Conductivity

**Francisco Alfredo Garcia-Pastor [1,*], Josué Benjamín Montelongo-Vega [1,4], Marco Vinicio Tovar-Padilla [2], María Antonia Cardona-Castro [1] and Jaime Alvarez-Quintana [2,3,*]**

[1] Centro de Investigación y de Estudios Avanzados del Instituto Politécnico Nacional Unidad Saltillo. Avenida Industria Metalúrgica No. 1062. Parque Industrial, 25900 Ramos Arizpe, Coahuila, Mexico; jmontelo@ternium.com.mx (J.B.M.-V.); antonia.cardona@cinvestav.edu.mx (M.A.C.-C.)

[2] Centro de Investigación en Materiales Avanzados S. C. Unidad Monterrey, Alianza Norte # 202. Autopista Mty-Aeropuerto Km.10. 66600 Apodaca, Nuevo León, México; opira666@gmail.com

[3] Genes-Group of Embedded Nanomaterials for Energy Scavenging, CIMAV-Unidad Monterrey, 66600, Apodaca, Nuevo León, Mexico

[4] Ternium Mexico S.A. de C.V, Planta Pesquería, Carretera a Pesquería Km. 15, Ejido La Victoria, 66650 Los Ramones, N.L.Mexico.

* Correspondente: francisco.garcia@cinvestav.edu.mx (F.A.G.-P.); jaime.alvarez@cimav.edu.mx (J.A.-Q.)

Received: 19 September 2020; Accepted: 29 October 2020; Published: date

---

Determination of the interface physical properties for actual surfaces such as real area in contact, the number of contact points per unit area, and the average thickness of the void gaps via image analysis, as well as calculation of the thermal contact resistance $R_{TCR}$. Determinations of the materials properties (Cu and Nb) such as electron mean free path, density of states, Fermi velocity, and transmission coefficient, as well as calculation of the interfacial thermal resistance $R_{ITR}$. Detailed discussion of the EDMM and TCR approaches, as well as calculations of the minimum thermal conductivity.

## Experimental Procedure

The phase diagram of binary Cu-Nb shows that this system is immiscible, and no intermixing should occur in the equilibrium state [1]. This is due to the difference in crystal structure between Cu and Nb, and the large atomic radii mismatch between the constituent atoms. Therefore, such materials are good candidates for the development of bulk nanolamellar Cu-Nb composites for robust thermal barrier applications. The starting materials consist of single-phase laminates of commercial purity Cu and Nb which are degreased, wire-brushed, and stacked. Iterating these steps increases the number of layers exponentially while decreasing layer thickness. A schematic representation of the general ARB process is shown in Figure S1a. ARB processing was carried out in a rolling mill with a maximum separating force of 20 metric tons, equipped with D2 steel rollers of 10 cm in diameter, following the procedure as described in reference [2]. Before stacking, surfaces of Cu-Nb-Cu sheets of 500, 1000 and 500 microns thick, respectively, were brushed and cleaned using acetone in an ultrasonic bath. Then, stacks were preheated in an inert atmosphere furnace at 700 °C for 4 min, and the rolling linear speed was set at $5 \times 10^{-3}$ m/s. After each ARB pass, the bonded laminate was cut transversally in half using a mechanical shear. The two halves were then subjected to a repetitive process of cleaning, stacking and rolling until obtaining the multilayers with ultrahigh content of interfaces.

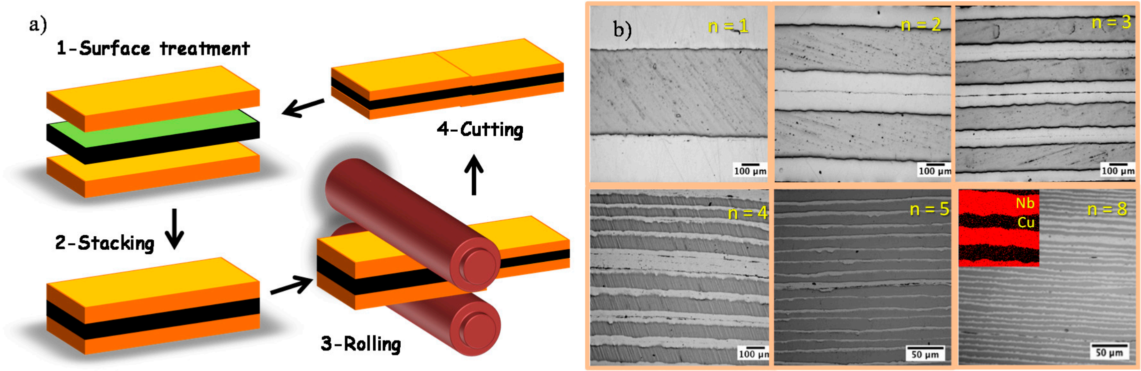


**Figure S1.** (**a**) Schematic of the ARB process, and (**b**) Optical images for samples with a different number of ARB iterations. Inset shows the EDS chemical analysis of the Nb-Cu multilayers.

In Figure S1b, optical images of the multilayers after eight sequential ABR process are shown. Evidently, after each ABR stage, the number of interfaces $N$ is increased following the power law $N = 2^n$, where $n$ stands for the process number. Based on this, the optical image with $n = 8$ presents a transversal section of the sample containing 256 interfaces, which is a considerable number of interfaces. The inset shows the EDS chemical analysis of the multilayers, Cu and Nb were identified as alternating layers. Although ARB was applied to several different bimetal systems [3-6], the Cu-Nb system offers low solid solubility between the two phases. These characteristics result in excellent microstructural stability during ARB processing, allowing production of bulk metallic nanolaminates. Likewise, Figure S2 shows SEM images for samples with $n = 11$, $n = 12$ and $n = 13$ iterations, which theoretically correspond to $N = 2048$, $N = 4096$ and $N = 8192$ interfaces, respectively. Well-defined interfaces can be seen formed by continuous layers of some nanometers in thickness, e.g., in sample with $n = 13$, a period below 40 nm is identified. Insets present HRTEM images at the interfaces.

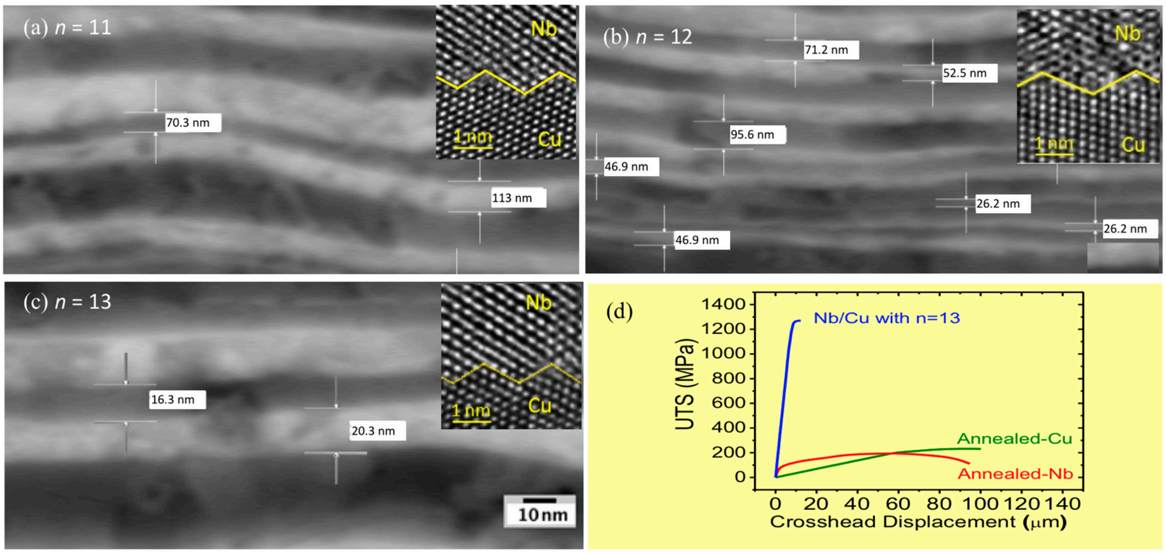


**Figure S2.** (**a**) TEM image of the Nb-Cu multilayers with $n = 11$, (**b**) $n = 12$ and (**c**) ($n = 13$ ARB iterations. (**d**) UTS for sample with $n = 13$ ARB iterations as compared with their annealed constituents. Insets shows HRTEM images of the multilayers at the interface.

## Thermal conductivity measurements of samples

Thermal response measurement of the samples was carried out via the hot-plate method. In such technique, the sample is installed between the hot plate (heater element) and cold plate (Aluminum heat sink), as it is shown in Figure S3. The heater element is placed on Silica aerogel tiles to reduce the heat leakage by conduction. Additionally, the whole system is put inside of a conventional

vacuum system to avoid heat losses by convection. A radiation shield is also used in order to protect the equipment from thermal fluctuations coming from the environment. Furthermore, silver paste is used to enhance thermal contact between sample surfaces and heater element as well as cold plate. Then, once steady state conditions have been established at room temperature, a DC electrical current with regular increments is applied into a 1 kΩ resistive heater by using the Keithley 6221 AC/DC power supply (company, city, state, country). Measurements are performed until steady state is accomplished for each DC electrical current level applied. This action will cause several temperature rises $\Delta T$ on the sample which are sensed via micro-thermocouples embedded into the hot and cold plates, respectively. Temperatures from thermocouples are logged by using a data acquisition module from national instruments model NI-9213. In this way, a set of $\Delta T$ and their corresponding heat fluxes $Q$ are obtained, then finally the transfer curve $Q$–$\Delta T$ can be plotted.

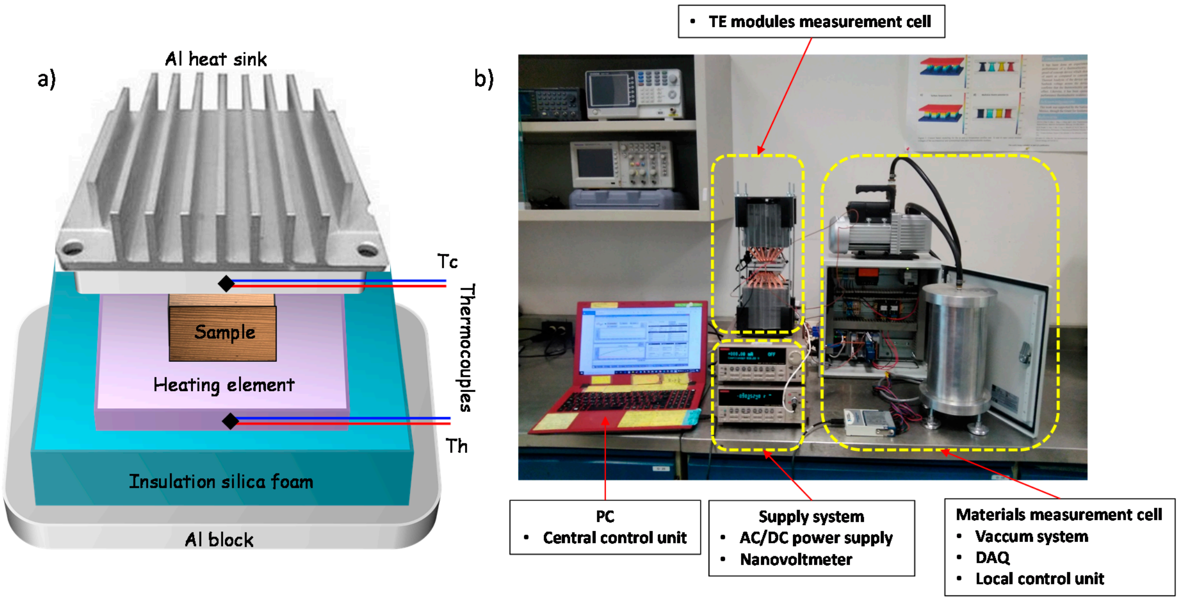


**Figure S3.** (**a**) Illustrative and (**b**) actual experimental set up for thermal conductivity measurements.

To verify the correct performance of the system, before each ARB sample measurement, a reference sample of $SiO_2$ was used for thermal conductivity system calibration. System calibration is performed via thermal conductivity measurement of a reference sample consisting of 1.1 cm x 1.1 cm x 1 mm of a corning glass slide. Figure S4 shows the $\Delta T$–$Q$ transfer curve for $SiO_2$ corning slide. By using the relation $k = L/RA$, the thermal conductivity of the sample is calculated. Here $L$ is the sample thickness, $A$ the area, and $R$ the thermal resistance which is estimated from the slope of the $\Delta T$–$Q$ curve. A value of κ ~ 1.37W/mK is obtained for $SiO_2$ calibration sample. Such result is in good agreement with previously reported values for $SiO_2$ [7]; hence, the system is reliable for measuring thermal conductivity. Moreover, to ensure measurement repeatability, each measurement was performed at least 5 times, results shown in Figure S4 are the average of the whole set of measurements for the $SiO_2$ sample, whereas Figure S5 shows the results for ARB sample with n = 13.

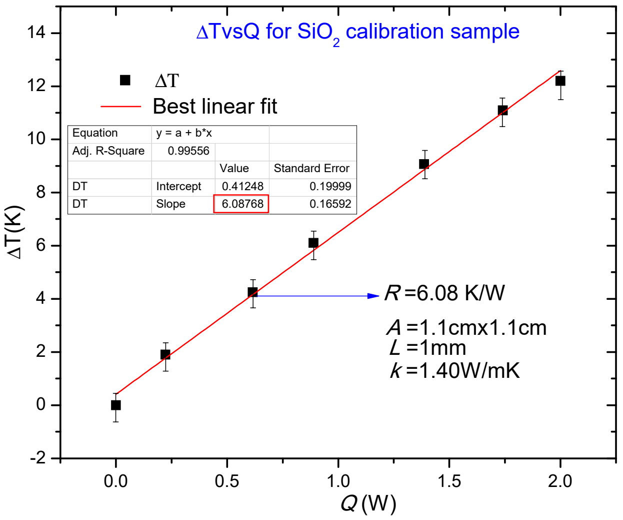


**Figure S4.** $\Delta T$–$Q$ transfer curve for reference sample.

By a similar procedure, the thermal conductivity of the multilayer samples from $n = 1$ to $n = 13$ was calculated. Figure S5 shows the result for sample with $n = 13$ iterations. As you can see, a value of κ~0.81W/mK is obtained.

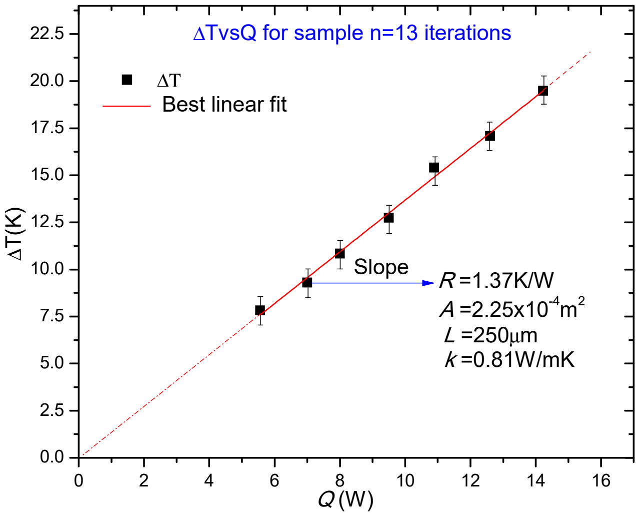


**Figure S5.** $\Delta T$–$Q$ transfer curve for sample with $n = 13$ iterations.

**Determination of parameters for modeling $R_{TCR}$ and calculations**

In order to determine $\alpha, \delta_i$, and $n$, we analyze the interfaces of the two contacting surfaces using SEM images, as well as the specialized Digital Micrograph GATAN software (Gatan Microscopy Suite Software, version 3, Gatan, Inc., Pleasanton, CA, USA). With the aim to show the general procedure employed, an arbitrary sample with $n = 2$ iterations is used as example. However, it is worth mentioning that all samples with period length in the mm range, i.e., $1 \lesssim n \lesssim 6$ were analyzed following the next procedure. Figure S6 shows an illustrative scheme of the parameters estimated so as to determine $\alpha$ which is the squared root of the ratio of the real area of contact $Ac$ to the total contact area $A$. $\delta_i$ is a parameter which expresses the equivalent idealized gap thickness in terms of the average heights $\overline{Z_{\iota xJ}}$, and $\overline{Z_{\iota yJ}}$ of the voids of actual surfaces in the $x$ and $y$ direction, respectively. Additionally, the number of contact points per unit area $n$ is obtained by dividing the number of contacts on each pair of profiles in the $x$ and $y$ directions, i.e., $n_{xi}$ and $n_{yi}$ over an area defined by $l_x l_y$.

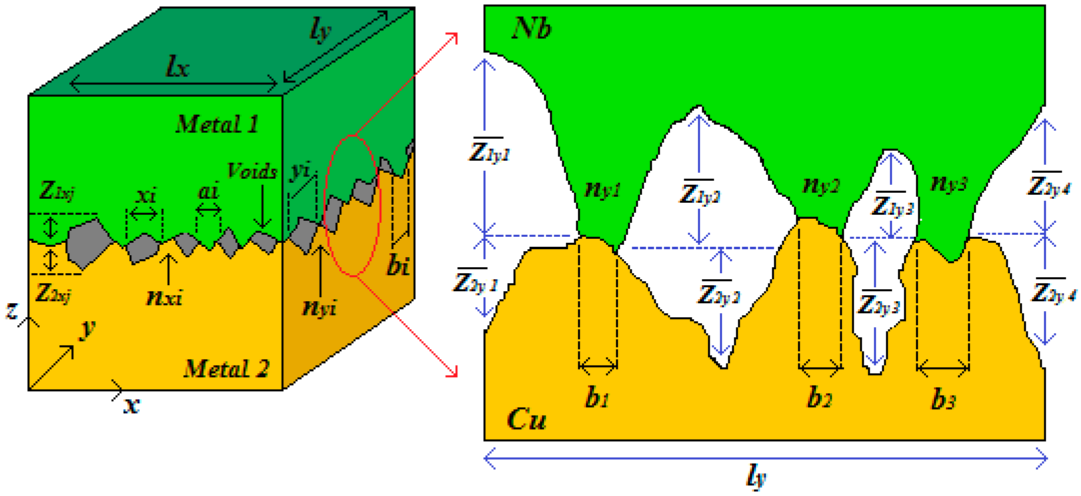


**Figure S6.** Illustrative model of an interface between two contacting metallic surfaces for samples with period lengths in the mm range, i.e., $1 \lesssim n \lesssim 6$. In the figure, $n_{xi}$, $a_i$, $x_i$ and $n_{yi}$, $b_i$, $y_i$, represent the number of contact points, size of the contact points and size of the voids in the $x$ and $y$ direction, respectively.

Clearly, Figure S6 shows an illustrative and idealized representation of actual interfaces. For most contacts, the height of the void is small compared with is width. Under this condition, it is reasonable to neglect both radial and convection heat conduction in the voids, thus, the thermal contact resistance $R_{TCR}$ associated with these type of interfaces can be estimated using the method proposed by W.M. Rohsenow [8] as

$$\frac{1}{R_{TCR}} = \frac{4.26\alpha\sqrt{n} + \left(\frac{k_f}{\delta_1 + \delta_2}\right)\left[(1-\alpha^2)C + 1.1\alpha\left(\frac{1}{k_1} + \frac{1}{k_2}\right)\right]}{(1-\alpha^2)\left[1 - \left(\frac{k_f}{\delta_1 + \delta_2}\right)\left(\frac{\delta_1}{k_1} + \frac{\delta_2}{k_2}\right)\right]C} \quad (1)$$

This expression for $R_{TCR}$ is the sum of two terms. The first term represents the heat flow through the metallic contact, and the second term with the square brackets in the numerator represents the heat flow across the voids. $k_i$ represents the thermal conductivity of the metals forming the interface, and $k_f$ the thermal conductivity of the fluid in the void. Likewise, $\alpha$ is the squared root of the ratio of the real area of contact $Ac$ to the total contact area $A$ which is approximately

$$\alpha = \left(\frac{A_c}{A}\right)^{1/2} \quad (2)$$

Figure S7a shows the period length as function of the iteration number $n$ for the ARB Cu-Nb composites. It can be seen that period length approximately spans over three scales; mm range for $1 \lesssim n \lesssim 6$, µm range for $7 \lesssim n \lesssim 10$, and nm range for $11 \lesssim n \lesssim 13$. Thus, only samples for $n \gtrsim 11$ must experience significant ITR effects. Therefore, the drastic reduction observed in κ for the ARB Cu-Nb composites with $n \lesssim 10$ is linked to the TCR. Inset in Figure 7a shows an optical image for sample with $n = 1$ iteration, contact points and voids are evident at the interfaces. Moreover, Figures 7b, c and d show SEM images for samples with $n = 2$, $n = 4$ and $n = 6$ iterations, respectively. Clearly, when pressure on the interface is increased via ARB process, the points in contact are deformed and they increase both in size and number, as highlighted by the red arrows in the images for samples with $n = 2$ and $n = 4$. Hence, the interfaces between metals become gradually in total contact because of the reduction of the surface's roughness, as shown for sample with $n = 6$.

To gain a quantitative understanding of these results, we have used the method proposed by W.M. Rohsenow [8]. In such an approach, the height of the void must be small compared with its width so as to neglect both radial and convection heat conduction in the voids.

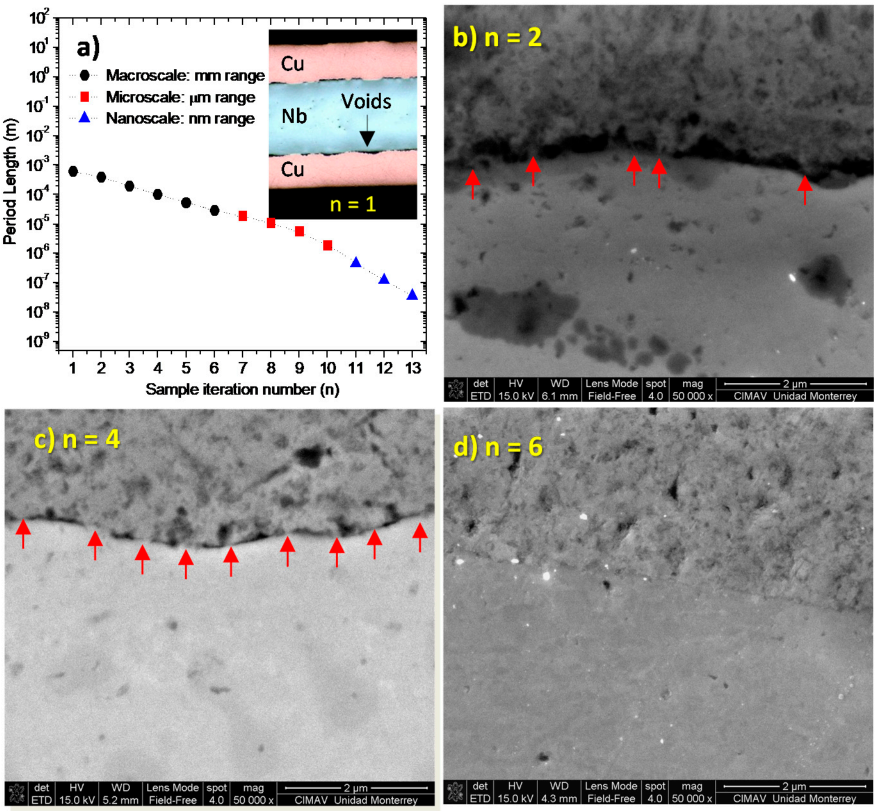


**Figure S7.** (**a**) Period length vs. iteration number *n* for the ARB Cu-Nb composites, and (**b**) *n* = 2, (**c**) *n* = 4, (**d**) *n* = 6 present the interface evolution.

## Determination of the real area of contact *Ac*

The real area of contact *Ac* is directly obtained by adding the projected width on the plane of contact of all contact points for each directional profile and by multiplying the total contact width in one direction by the total contact width in the perpendicular direction. Thus, calling the width of the contacts in the *x* direction $a_1, a_2, \ldots, a_n$, and in the *y* direction $b_1, b_2, \ldots, b_n$ (see Figure S6), the real area of contact and the total contact area *A* are given by

$$A_c = \sum_{i=1}^{n_{xn}} \sum_{j=1}^{n_{ym}} a_i b_j \quad (3)$$

$$A = l_x l_y \quad (4)$$

where $l_x$ and $l_y$ are the total lengths of the recorded profiles in each direction. Figure S8 shows the length estimations for the $a_i$ and $b_i$ for an interface of the sample with $n = 2$ iterations.

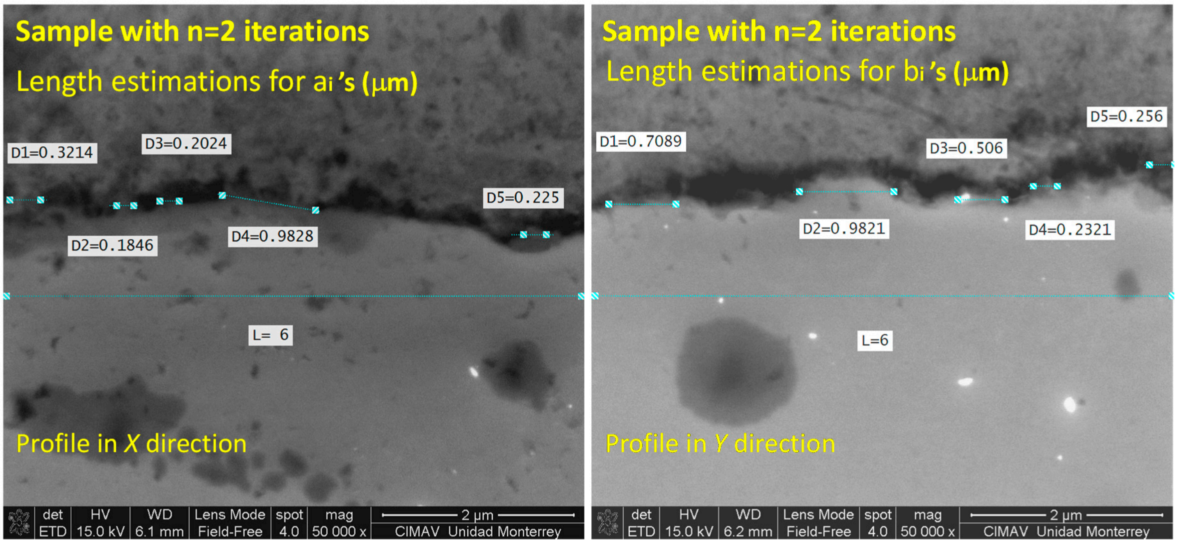


**Figure S8.** Size of the contact points in the $x$ and $y$ directions, respectively.

By using Equations (3) and (4), as well as the data extracted from Figure S8, a value of $\alpha \sim 0.021$ was calculated along with Equation (2).

**Determination of the $\delta_i$ parameter**

Furthermore, in Equation (1), $\delta_i$ is a parameter which expresses the equivalent idealized gap thickness in terms of the average thicknesses $\overline{Z_{ixj}}$, and $\overline{Z_{iyj}}$ of the voids of actual surfaces in the $x$ and $y$ directions, respectively, as shown in Figure 6. The subscript $i$ refers to metal 1 or 2, and $j$ refers to the number of the void. Thus, the following expression for $\delta_i$ is defined by

$$\delta_i = \frac{Z_i}{\left(1 - \frac{k_f}{k_i}\right)} \quad (5)$$

where $Z_i$ is then calculated using the relations: $Z_1 = \overline{Z_{1yi}} + \overline{Z_{1xi}}(1 - \beta_y)$ and $Z_2 = \overline{Z_{2yi}} + \overline{Z_{2xi}}(1 - \beta_x)$. Figure S9 shows the measurements of the heights $\overline{Z_{ixj}}$, and $\overline{Z_{iyj}}$.

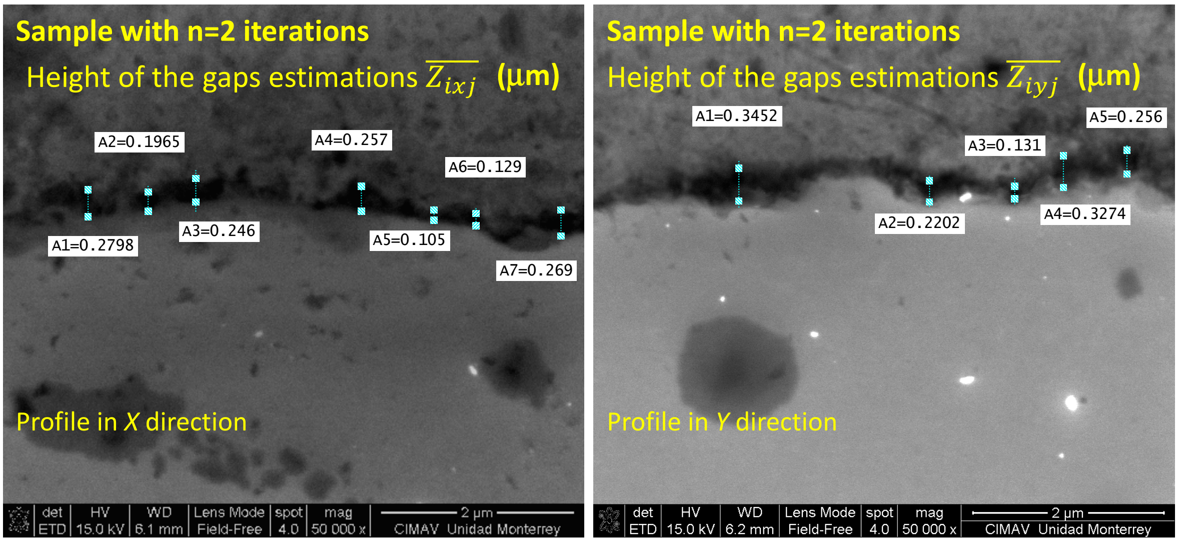


**Figure S9.** Average heights $\overline{Z_{ixj}}$, and $\overline{Z_{iyj}}$ of the voids of actual surfaces in the $x$ and $y$ directions, respectively.

Furthermore, the parameters $\beta_x$ and $\beta_y$ can be estimated by

$$\beta_y = \frac{\sum_i y_i}{l_y} \quad (6)$$

$$\beta_x = \frac{\sum_i x_i}{l_x} \quad (7)$$

The summation extending over all the void segments $x_i$ and $y_i$ of the $l_x$ and $l_y$ profiles, respectively. Figure S10 shows the measurements of the width of the voids in each direction. By using Equations (6) and (7), as well as the data extracted from Figure 9 and Figure 10, values of $\delta_1 \sim 1.49x10^{-7}m$ and $\delta_2 \sim 8.93x10^{-8}m$ were calculated along with Equation (5).

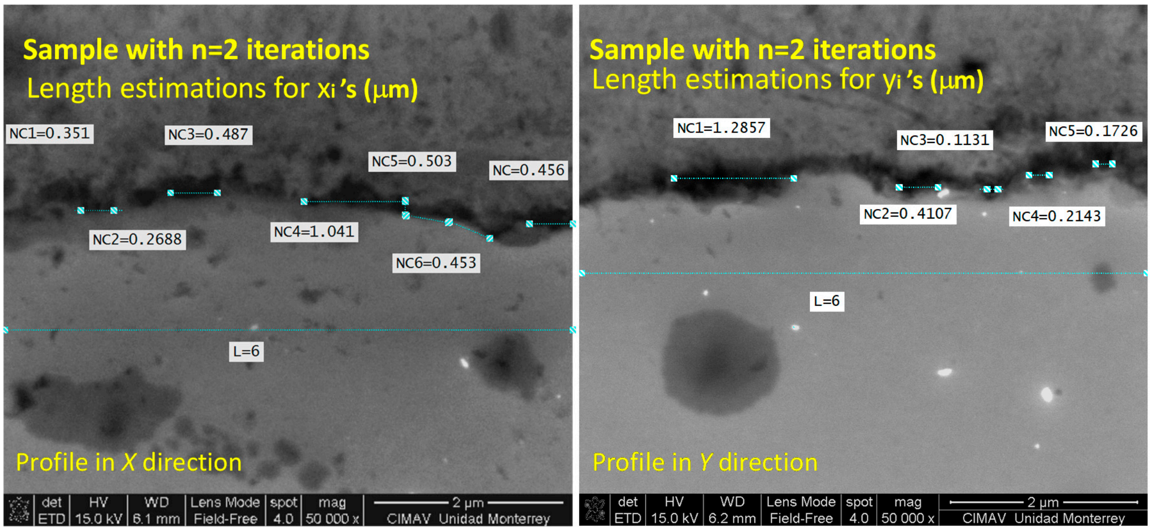


**Figure S10.** Size of the voids in the $x$ and $y$ directions, respectively.

**Determination of the number of contact points per unit area *n***

Moreover, the number of contact points per unit area $n$ is obtained by counting the number of contacts on each pair of profiles in the $x$ and $y$ directions, i.e., $n_{xi}$ and $n_{yi}$. Since the two directions are perpendicular to one another, one deduces that over an area $l_x l_y$, an approximation for $n$ is

$$n \cong \frac{n_x n_y}{l_x l_y} \quad (8)$$

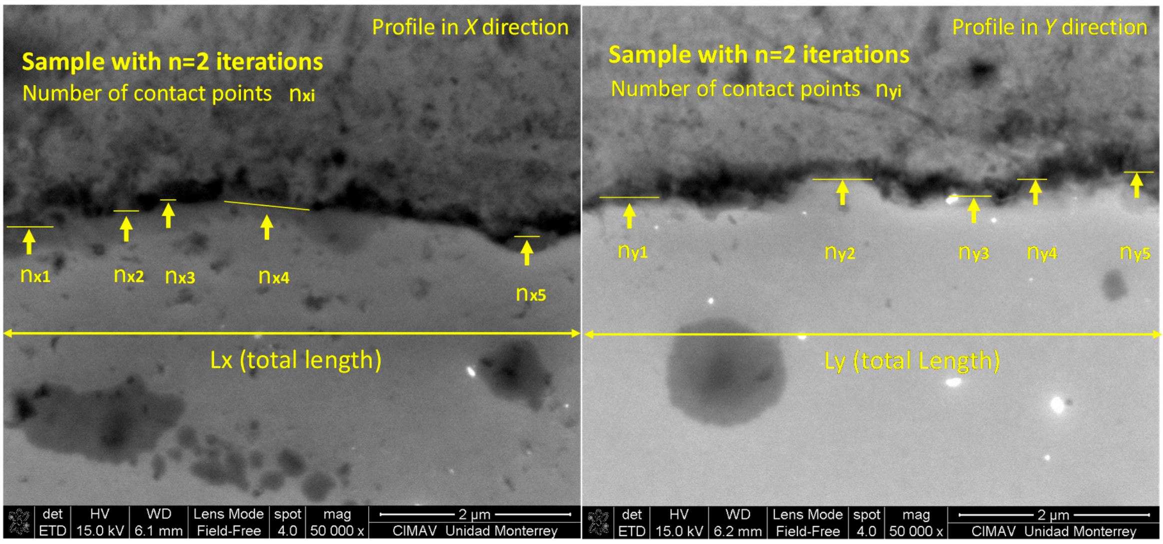


**Figure S11.** Number of contact points per unit area $n$.

By using Equation (8), as well as the data extracted from Figure 11, a value of $n{\sim}6.9x10^{11}m^{-2}$ was calculated.

The term $C$ is a factor which is only used to compact Equation (1), and it is given by

$$C = \frac{1 + 4.26\sqrt{n}\left(\frac{\delta_1}{\alpha}\right)}{k_{1(Nb)}} + \frac{1 + 4.26\sqrt{n}\left(\frac{\delta_2}{\alpha}\right)}{k_{2(Cu)}} \tag{9}$$

By applying the values obtained for $\alpha$, $n$ and $\delta_i$, a value of $C{\sim}0.573$ is calculated. Here, $k_{1(Nb)}$ and $k_{2(Cu)}$ stand for the bulk thermal conductivity of the Nb and Cu, respectively.

Finally, with the parameters $\alpha$, $n$, and $\delta_i$, and the factor $C$, the magnitude of $R_{TCR}$ is calculated by using Equation (1). A summary of the parameters used for calculations of the thermal contact resistance of the sample with $n = 2$ iterations is shown in Table S1.

**Table S1.** Parameters used for sample with $n = 2$ iterations.

| $\alpha$ | $\delta_1$ | $\delta_2$ | $n$ | $k_{1(Nb)}$ | $k_{2(Cu)}$ | $k_{f(air)}$ | $R_{TCR}$ |
|---|---|---|---|---|---|---|---|
| 0.021 | $1.49 \times 10^{-7}m$ | $8.93 \times 10^{-8}m$ | $6.9 \times 10^{11}m^{-2}$ | $54W/mK$ | 400 W/mK | 0.02 W/mK | $4.7 \times 10^{-6}m^2K/W$ |

An effective thermal conductivity value of $k{\sim}13.5W/mK$ was estimated using the value of $R_{TCR} = 4.7 \times 10^{-6}m^2K/W$ for the sample with $n = 2$ iterations.

The above procedure was performed for samples with $1 \lesssim n \lesssim 6$ iterations; here, only the procedure for a sample with $n = 2$ is illustrated.

The problem with Equation (3) is related with the function $f(\alpha)$, which is defined by

$$f(\alpha) = \frac{Y_0(2.20\alpha)J_1(3.83\alpha) - Y_1(2.20\alpha)J_0(3.83\alpha)}{1.75Y_0(2.20\alpha)J_1(3.83\alpha) - Y_1(2.20\alpha)J_0(3.83\alpha)} \tag{10}$$

$Y_i$ and $J_i$ are the Bessel functions of the first and second kind. For practical purposes, the ratio of the squared root of the real area of contact to the total contact area must be $\alpha \lesssim 0.1$. Under this condition, $f(\alpha) \approx 1$, otherwise, $f(\alpha)$ drops drastically well below 1. The physical meaning of $f(\alpha) \ll 1$ implies that the real area of contact to the total contact are very similar, thus $\alpha \approx 1$, i.e., no more voids or a very high contact points density; hence, the model fails in such a situation. To overcome such condition, the first term in Equation (1), with the square brackets in the numerator, can be neglected because it represents the heat flow across the voids. By using such mathematical artifice, Equation (1) becomes independent of $f(\alpha)$, and the model can be applied for samples with periods at microscale where the interfaces have much better contact, i.e., $7 \lesssim n \lesssim 10$.

**Determination of parameters for modeling $R_{ITR}$ and calculations**

In the EDMM approach, the interfacial thermal resistance is given by [9]

$$\frac{1}{R_{ITR}} = \frac{1}{4}T_{1\to2}Z_1 \tag{11}$$

where $Z_i = C_{ei}v_{Fi}$, and $C_{ei}$ is the electronic heat capacity of the metal in side *i*, as well as $v_{Fi}$ being the electron Fermi velocity. Besides, $T_{1\to2}$ is the transmission coefficient which is given by

$$T_{1\to2} = \frac{Z_2}{Z_1 + Z_2} \tag{12}$$

Here, electronic heat capacity $C_{ei}$ is

$$C_{ei} = \frac{\pi^2 k_B^2 D_i}{3}T \tag{13}$$

and $D_i$ denotes the density of states of the metal in side *i*, which is given by

$$D_i = \frac{(2m^3)^{1/2}}{2\pi^2\hbar^3}E^{1/2} \tag{14}$$

Furthermore, the electron velocity on Fermi surface is

$$v_{Fi} = \left(\frac{2E_F}{m}\right)^{1/2} \tag{15}$$

Moreover, the mean speed of all the electrons can be calculated from the Fermi velocity by

$$\bar{v}_{Fi} = \left(\frac{3v_{Fi}^2}{5}\right)^{1/2} \tag{16}$$

Hence, the mean free path in turn is

$$\bar{\lambda_i} = \frac{3k_i}{C_{ei}\bar{v}_{Fi}} \tag{17}$$

By using Equations (13) to (17), the material properties for Cu and Nb were estimated. Data are shown in Table S2.

**Table S2.** Calculated parameters for Cu and Nb using the free electron model.

| Material | $D_i$ | $E_{Fi}$ | $v_{Fi}$ | $\bar{v}_{Fi}$ | $C_{ei}$ | $m$ | $\bar{\lambda_i}$ |
|---|---|---|---|---|---|---|---|
| Cu | $1.17 \times 10^{47}$ m$^{-3}$ | 7.5 eV | $1.63 \times 10^6$ m/s | $1.26 \times 10^6$ m/s | 10948.55 J/m$^3$K | m$_o$ | 86 nm |
| Nb | $2.9 \times 10^{47}$ m$^{-3}$ | 5.0 eV | $9.01 \times 10^5$ m/s | $6.97 \times 10^5$ m/s | 27253.72 J/m$^3$K | m$_o$ | 8 nm |

Additionally, the interfacial thermal resistance and the transmission coefficient were estimated via Equations (11) and (12), respectively, values are shown in Table S3.

**Table S3.** Calculated data for the interface thermal resistance between copper and niobium interfaces.

| $T_{1\to2}$ | $R_{ITR(1\to2)}$ | $R_D$ | $R_B$ |
|---|---|---|---|
| 0.42 | $9.7 \times 10^{-11}$ m$^2$·K/W | $9.7 \times 10^{-11}$ m$^2$·K/W | $4.07 \times 10^{-11}$ m$^2$·K/W |

By analyzing the results, evidently, electrons move ballistically from the copper side to the niobium side, where then they scatter diffusively depending on the surface roughness. The effective thermal interface resistance is thus the sum of the contribution of both the electrons ballistically crossing the Cu–Nb interface ($R_{ITR(1\to2)} = R_D$), and those diffusively scattering in Niobium ($R_B$). Therefore, the effective ITR is given by

$$\frac{1}{R_{eITR}} = \frac{1}{R_D}(\beta) + \frac{1}{R_B}(1-\beta) \tag{18}$$

where $\beta = e^{(-d/\lambda_e)}$ is a term derived from ballistic diffusive equations [10] and gives the exponential decay of heat flux across interface, $d$ is half the period thickness and $\lambda_e$ is an effective mean free path of the electrons in the Cu–Nb system seen as an effective medium. Besides, the ballistic component $R_B$ is related to the diffusive component $R_D$ through

$$\frac{1}{R_B} = \frac{1}{T_{1\to2}R_d} \tag{19}$$

Table S4 shows the obtained results via Equations (20) and (21) for the $R_{eITR}$.

**Table S4.** Effective thermal interface resistance calculations for samples with $n = 11$, $n = 12$ and $n = 13$ iterations.

| Sample | $\beta$ | $R_{eITR}$ |
|---|---|---|
| $n = 11$ | 0.0015 | $9.68 \times 10^{-11}$ m$^2$·K/W |
| $n = 12$ | 0.165 | $7.91 \times 10^{-11}$ m$^2$·K/W |
| $n = 13$ | 0.56 | $5.46 \times 10^{-11}$ m$^2$·K/W |

**Minimum thermal conductivity calculations**

Here, following Cahill and co-workers, we use "ultra-low" to describe a κ value lower than what is predicted by the minimum κ model developed by Cahill et al. Therefore, the existence of a lower limit to the thermal conductivity of disordered crystals based on the idea that lattice vibrations in those solids are essentially the same as those of an amorphous solid can be estimated by

$$k_{min} = \left(\frac{\pi}{6}\right)^{1/3} k_B n^{2/3} \sum_i v_i \left(\frac{T}{\theta_i}\right)^2 \int_0^{\theta_i/T} \frac{x^3 e^x}{(e^x - 1)^2} dx \quad (20)$$

Equation (20) can be reduced to

$$k_{min} = 0.403 k_B n^{2/3} (2v_T + v_L) \quad (21)$$

where $v_T$ and $v_L$ are the transversal and longitudinal speeds of the sound, respectively, $n_a$ is the number of density of atoms, and $k_B$ the constant of Boltzmann. For comparison, Table 1 shows the minimum thermal conductivity values predicted by Equation (21), as well as the reported experimental thermal conductivity for amorphous bulk materials. Clearly, thermal conductivity value of 0.81W/mK at room temperature which is even lower than the amorphous lattice limit for the Cu-Nb thin film system and is determined to be well below that of the amorphous dielectrics like $Al_2O_3$ or $SiO_2$.

**Table S5.** Predicted values for the lower limit to thermal conductivity of amorphous materials.

| Sample | $n$ ($10^{28}$ m$^{-3}$) | $v_T$ (m/s) | $v_L$ (m/s) | $K_{min}$ (W/mK) | $K_{exp}$ (W/mK) |
|---|---|---|---|---|---|
| $SiO_2$ | 6.63 | 3740 | 5980 | 1.21 | 1.35 |
| $Al_2O_3$ | 10.89 | 5800 | 9900 | 2.71 | 2.76 |
| Cu | 8.47 | 3720 | 4720 | 1.3 | - |
| Nb | 5.56 | 2092 | 5068 | 0.76 | - |
| a-Cu/Nb system | | | | 0.95 | |
| Cu-Nb(n = 13) | | | | | 0.81 |

**Experimental interfacial thermal resistance estimations at nanoscale**

The interfacial thermal resistance at nanoscale was estimated from the experimental $\Delta T$–$Q$ transfer curves. The sample thermal resistance can be expressed as

$$RA = \left[\frac{d_1}{k_{Cu-film}} + \frac{d_2}{k_{Nb-film}}\right] N + R_{ITR} N \quad (22)$$

where terms in brackets represent the thermal resistance due to the Cu and Nb films, whereas the last term represents the thermal resistance due to the total number N of interfaces. Evidently, the interfacial thermal resistance due to a single Cu-Nb interface is given by

$$R_{ITR} = \frac{RA}{N} - \left[\frac{d_1}{k_{Cu-film}} + \frac{d_2}{k_{Nb-film}}\right] \quad (23)$$

It is clear that Cu and Nb films are not monocrystalline; in this sense, it is well known that energy carriers experience severe size confinement and scattering at grain boundaries as the mean free path accomplish or even overcome the sample size [11].

For the sake of simplicity, by using a previously reported model [12] we estimated the size-dependent electron thermal conductivity in Cu($k_{Cu\text{-}FILM}$) and Nb($k_{Cu\text{-}FILM}$) films. Results are shown in Figure 12, and to confirm the validity of the modeling results, experimental thermal conductivity values for Cu were obtained from the existing literature [13,14], whereas size-dependent thermal conductivity data for Nb are completely absent in literature. However, due to the modeling results being consistent with the existing data for Cu, we assumed that the experimental data for Nb can be predicted with reasonable uncertainty. Based on this, the thermal conductivity data obtained for

polycrystalline films of 20nm thick of Cu, and 30nm thick of Nb are $k_{Cu\text{-}FILM}$=13.7W/mK, and $k_{Nb\text{-}FILM}$=0.83W/mK, respectively.

By substituting such values into $R_{ITR} \times N = R \times A-[(d/k_{Cu\text{-}FILM}) + (d/k_{Nb\text{-}FILM})] \times N$, for N = 8192 (sample *n* = 13), we get:

$R_{ITR} \times N=(1.37 \times 2.25 \times 10^{-4}\ m^2\cdot K/W)-[(20\ nm/13.7\ W/mK) + (30\ nm/0.83\ W/mK)] \times 8192=1.64 \times 10^{-7}\ m^2\cdot K/W$; therefore, $R_{ITR}= (1.649 \times 10^{-7}/8192) m^2\cdot K/W = 2.1 \times 10^{-11}\ m^2\cdot K/W$; such value corresponds to the value of the interfacial thermal resistance for sample *n* = 13. By a similar procedure, the interfacial thermal resistance values were obtained for sample *n* = 11, and *n* = 12 from the experimental data of the corresponding slope of the Q vs ΔT plot.

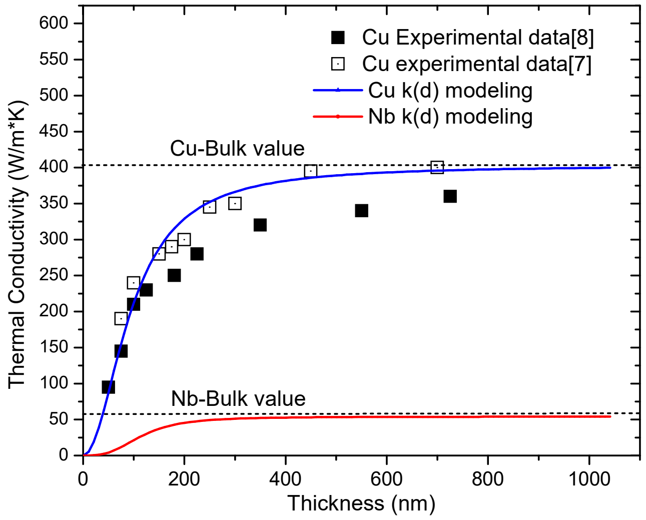


**Figure S12.** Size-dependent thermal conductivity for Cu and Nb polycrystalline films. Solid lines represent the results obtained from model reported in reference [12], whereas solid points represent the existing experimental data for Cu.

**Uncertainty assessment**

In connection to the uncertainty sources, a common method developed by Moffat [15] was used in the present research for estimating the overall uncertainty in the measurements. This method is based on the specification of the uncertainties in the various primary experimentally measured parameters. For instance, If the uncertainty present in $Q$–$\Delta T$ plots is a function of several measured parameters ($P_1$, $P_2$, …, $P_n$) each with a corresponding uncertainty ($\Delta P_1$, $\Delta P_2$, …, $\Delta P_n$), an overall uncertainty on the slope of the ($\Delta S$) of the $Q$–$\Delta T$ plots using the method of Moffat can be estimated:

$$\Delta S_{total} = \left[\left(\frac{\partial S}{\partial P_1}\right)^2 \Delta P_1 + \left(\frac{\partial S}{\partial P_2}\right)^2 \Delta P_2 + \left(\frac{\partial S}{\partial P_3}\right)^2 \Delta P_3 + \cdots \left(\frac{\partial S}{\partial P_n}\right)^2 \Delta P_n\right]^{1/2} \tag{24}$$

Considering that, the parameters $P_i$ experience small perturbations $\Delta P_i$, the partial derivatives can be approximated as:

$$\frac{\partial S}{\partial P_i} = \frac{S(P_i + \Delta P_i) - S(P_i)}{\Delta P_i} \sim \frac{\Delta S}{\Delta P_i} \tag{25}$$

The fitting error $\Delta S_i$ was performed assuming a *t*-distribution due to the small number of $Q$-$\Delta T$ points ($Q$,$\Delta T$) taken at each measurement, below 10 points. This type of error including noise source errors $\Delta S_{noise}$ only accounts for the random error in the experiment and was simply added to the systematic error found from a sensitivity analysis.

Table S6 summarizes the uncertainty values for each parameter that was varied in the analysis, the uncertainty in the slope of a typical $Q$–$\Delta T$ measurement can be determined based on the parameters' outline.

**Table S6.** Summary of typical uncertainty of measuring parameters.

| **Parameter $P_i$ (Error Source)** | **Nominal Value $P_i$ at 300K** | **Typical Uncertainty $\Delta P_i$** | $\frac{\partial S}{\partial P_i}$ |
|---|---|---|---|
| Area(mm$^2$) | $225 \times 10^{-6}$ m$^2$ (0.015 mm × 0.015 mm) | $\pm 5 \times 10^{-6}$m$^2$ | $\sim \Delta S/\Delta A$ |
| Length(mm) | $250 \times 10^{-6}$m | $\pm 10 \times 10^{-6}$m | $\sim \Delta S/\Delta L$ |
| Temperature (Each Measurement) | Temperature Measurements (Each Measurement—*n* Measurements along the line) | ±1.5K | $\sim \Delta S/\Delta T$ |
| Instrumentation Noise Sources | | ±5μV/K | |
| Fitting Error | | ±2.5% | |

Using the formulation $\Delta S_{total}$, the uncertainty in the slope can be determined based on the overall system parameters outlined in the above table. The uncertainty was found to be $\Delta S_{total} \sim 6.56\%$ for a determined *Q*−*ΔT* plot. Hence, the uncertainty in the measurements performed in the present research does not go beyond 10%. Based on this, uncertainty bars were added to the measurements present in the main manuscript.

**References**


1. Okamoto, H. Cu-Nb (Copper-Niobium). *J. Phase Equilibria Diffus.* **2012**, *33*, 344, doi:10.1007/s11669-012-0051-y.
2. Saito, Y.; Utsunomiya, H.; Tsuji, N.; Sakai, T. Novel ultra-high straining process for bulk materials—development of the accumulative roll-bonding (ARB) process. *Acta Mater.* **1999**, *47*, 579–583, doi:10.1016/s1359-6454(98)00365-6.
3. Yasuna, K.; Terauchi, M.; Otsuki, A.; Ishihara, K.; Shingu, P. Formation of nanoscale Fe/Ag multilayer by repeated press-rolling and its layer thickness dependence of magnetoresistance. *Mater. Sci. Eng. A* **2000**, *285*, 412–417, doi:10.1016/s0921-5093(00)00680-8.
4. Knezevic, M.; Nizolek, T.; Ardeljan, M.; Beyerlein, I.J.; Mara, N.A.; Pollock, T.M. Texture evolution in two-phase Zr/Nb lamellar composites during accumulative roll bonding. *Int. J. Plast.* **2014**, *57*, 16–28, doi:10.1016/j.ijplas.2014.01.008.
5. Chang, H.; Zheng, M.; Gan, W.; Wu, K.; Maawad, E.; Brokmeier, H. Texture evolution of the Mg/Al laminated composite fabricated by the accumulative roll bonding. *Scr. Mater.* **2009**, *61*, 717–720, doi:10.1016/j.scriptamat.2009.06.014.
6. Hosseini, M.; Pardis, N.; Manesh, H.D.; Abbasi, M.; Kim, D.-I. Structural characteristics of Cu/Ti bimetal composite produced by accumulative roll-bonding (ARB). *Mater. Des.* **2017**, *113*, 128–136, doi:10.1016/j.matdes.2016.09.094.
7. Cahill, D.G.; Watson, S.K.; Pohl, R.O. Lower limit to the thermal conductivity of disordered crystals. *Phys. Rev. B* **1992**, *46*, 6131–6140, doi:10.1103/physrevb.46.6131.
8. Fenech, H.; Rohsenow, W.M. Prediction of Thermal Conductance of Metallic Surfaces in Contact. *J. Heat Transf.* **1963**, *85*, 15–24, doi:10.1115/1.3686003.
9. Gundrum, B.C.; Cahill, D.G.; Averback, R.S. Thermal conductance of metal-metal interfaces. *Phys. Rev. B* **2005**, *72*, 245426, doi:10.1103/physrevb.72.245426.
10. Gang, C. Ballistic-Diffusive Heat-Conduction Equations. *Phys. Rev.Lett.* **2001**, *86*, 2297–2300.
11. Mayadas, A.F.; Shatzkes, M. Electrical-Resistivity Model for Polycrystalline Films: the Case of Arbitrary Reflection at External Surfaces. *Phys. Rev. B 1* **1970**, *1*, 1382, doi:10.1103/PhysRevB.1.1382.
12. Jin, J.S.; Lee, J.S.; Kwon, O. Electron effective mean free path and thermal conductivity predictions of metallic thin films. *Appl. Phys. Lett.* **2008**, *92*, 171910, doi:10.1063/1.2917454.
13. Nath, P.; Chopra, K. L. Thermal conductivity of copper films. *Thin Solid Films* **1974**, *20*, 53–62, doi:10.1016/0040-6090(74)90033-9.
14. Kelemen, F. Pulse method for the measurement of the thermal conductivity of thin films. *Thin Solid Films* **1976**, 36, 199–203, doi:10.1016/0040-6090(76)90440-5
15. Moffat, R.J. Describing the uncertainties in experimental results. *Exp. Therm. Fluid Sci.* **1988**, *1*, 3–17, doi:10.1016/0894-1777(88)90043-x.
